# Motivating men who have sex with men to get tested for HIV

**DOI:** 10.1186/1742-4690-9-S1-P123

**Published:** 2012-05-25

**Authors:** Magaly M Blas, Luis Menacho, Isaac E Alva, Roberto Orellana

**Affiliations:** 1 Cayetano Heredia Peruvian University, Lima, Peru

## Introduction

Although men who have sex with men (MSM) have the highest HIV prevalence in Peru, they are underserved by traditional preventive programs. Interestingly, in Peru the Internet and cell-phones have emerged as a convenient tool to reach this population.

## Methods

From October 2010 to February 2011, we conducted eight focus groups with gay and non-gay identified MSM, and eight in-depth interviews with key informants in order to identify key features and preferences to be used to tailor culturally-appropriate behavioral messages that could be delivered through Internet and cell-phones to motivate MSM to get tested for HIV.

## Results

Participants reported that in order to motivate HIV testing among MSM, interventions need to be based on motivational messages that encourage participants to overcome the fear of getting tested. Messages should increase the HIV risk perception (of participants who do not consider themselves at risk) by eliciting risky situations usually experienced by MSM. Messages should emphasize the confidentiality, professionalism and respect of the personnel conducting the counseling and testing. A thorough explanation of the process of HIV testing, including information about the type of information will be collected, types of tests that will be available (rapid or ELISA), level of pain participants may feel, time to get the results back, and cost of the testing should be included. Additionally, detailed information about the steps participants have to follow if they test positive or negative should be provided. Messages should also contain detailed information about the venue where the test will be conducted in terms of type of clients who attend, location, hours of operation and personnel. Finally, stigmatizing and stereotyped messages or images about “being gay” should not be included, as they act as deterrents for getting tested.

## Conclusions

Interventions aimed at motivating HIV testing among MSM should include motivational messages that reduce the fear of getting tested and increase the risk perception of participants. They should also market the venue where the testing will be conducted, the professionals who will perform the tests, and the test itself. Stigmatizing messages or images should be avoided.

